# Convergence in Reflex Pathways from Multiple Cutaneous Nerves Innervating the Foot Depends upon the Number of Rhythmically Active Limbs during Locomotion

**DOI:** 10.1371/journal.pone.0104910

**Published:** 2014-08-29

**Authors:** Tsuyoshi Nakajima, Rinaldo A. Mezzarane, Sandra R. Hundza, Tomoyoshi Komiyama, E. Paul Zehr

**Affiliations:** 1 Department of Integrative Physiology, Kyorin University School of Medicine, Tokyo, Japan; 2 Rehabilitation Neuroscience Laboratory, University of Victoria, Victoria, BC, Canada; 3 Laboratory of Signal Processing and Motor Control, College of Physical Education, University of Brasília, Brasília, Brazil; 4 Motion and Mobility Rehabilitation Laboratory, University of Victoria, Victoria, BC, Canada; 5 Human Discovery Science, International Collaboration on Repair Discoveries (ICORD), Vancouver, BC, Canada; 6 Centre for Biomedical Research, University of Victoria, Victoria, BC, Canada; 7 Division of Sports and Health Science, Chiba University, Chiba, Japan; 8 Division of Medical Sciences, University of Victoria, Victoria, BC, Canada; Tokai University, Japan

## Abstract

Neural output from the locomotor system for each arm and leg influences the spinal motoneuronal pools directly and indirectly through interneuronal (IN) reflex networks. While well documented in other species, less is known about the functions and features of convergence in common IN reflex system from cutaneous afferents innervating different foot regions during remote arm and leg movement in humans. The purpose of the present study was to use spatial facilitation to examine possible convergence in common reflex pathways during rhythmic locomotor limb movements. Cutaneous reflexes were evoked in ipsilateral tibialis anterior muscle by stimulating (in random order) the sural nerve (SUR), the distal tibial nerve (TIB), and combined simultaneous stimulation of both nerves (TIB&SUR). Reflexes were evoked while participants performed rhythmic stepping and arm swinging movement with both arms and the leg contralateral to stimulation (ARM&LEG), with just arm movement (ARM) and with just contralateral leg movement (LEG). Stimulation intensities were just below threshold for evoking early latency (<80 ms to peak) reflexes. For each stimulus condition, rectified EMG signals were averaged while participants held static contractions in the stationary (stimulated) leg. During ARM&LEG movement, amplitudes of cutaneous reflexes evoked by combined TIB&SUR stimulation were significantly larger than simple mathematical summation of the amplitudes evoked by SUR or TIB alone. Interestingly, this extra facilitation seen during combined nerve stimulation was significantly reduced when performing ARM or LEG compared to ARM&LEG. We conclude that locomotor rhythmic limb movement induces excitation of common IN reflex pathways from cutaneous afferents innervating different foot regions. Importantly, activity in this pathway is most facilitated during ARM&LEG movement. These results suggest that transmission in IN reflex pathways is weighted according to the number of limbs directly engaged in human locomotor activity and underscores the importance of arm swing to support neuronal excitability in leg muscles.

## Introduction

Progress in understanding human movement indicates that the neuronal coordination for fore and hind limbs observed in quadrupedal locomotor systems is preserved in human locomotion [1]–[5]. This coordination may partly be mediated by coupled locomotor generator systems regulating rhythmic arm and leg movement [3], [4], [6]. One methodology for assessing this coordination in humans is to measure the modulation of segmental reflexes during rhythmic movement [2], [4].

Nerve or location-specificity of reflex amplitudes has been described in leg muscles during walking and leg cycling after activation of cutaneous nerves in the foot [4], [7]–[11] and in arm muscles during arm cycling after activation of cutaneous nerves in the hand [12]. The specific expression of location-specificity appears related to the functional role of cutaneous reflexes during locomotion [8], [9]–[13], [14]. Interestingly, despite some differences in the expression of reflex amplitudes based upon the nerve stimulated, there are some common features as well, suggesting the possibility of shared pathways [2].

Using spatial facilitation in a feline model, Labella and McCrea (1990) reported that cutaneous afferents from two different nerves converge onto common spinal interneurons (IN) to produce excitation and inhibition in functionally related groups of motoneurons [15]. Recently, by using a similar technique in humans, we found evidence for convergence in putative IN reflex circuits by simultaneous electrical stimulation of cutaneous nerves in the hand and foot during locomotor movement of all four limbs [16].

Although neural output from the locomotor generating systems for each arm and leg projects directly to each motoneuronal pool and indirectly through IN reflex networks [4], [17], [18], it is unknown whether these outputs modulate excitation of common reflex pathways.

Previous studies reported that during the rhythmic movement of “all four limbs”, the influence of the arms on reflex expression in the legs was superimposed on the dominant effect of the legs [19]–[23]. However, modulation of reflex amplitude in a muscle in a moving limb reduces the sensitivity for detecting influences of remote limb movement such as from the arm [20], [24]. That is, modulation of reflex activity from other limbs may be “swamped” by the dominant effect of moving the limb in which the test muscle resides. As an advanced protocol, therefore, we have used a three-limb (both arms and one leg) stepping paradigm as a form of “reduced” locomotion [23], [25]. This paradigm allows the test limb to remain in stationary and avoids possible occlusion of the modulation of reflex amplitude [23].

The purpose of the present study was to examine convergence in reflex effects following cutaneous stimulation of two different nerves of the foot during arm and leg three-limb stepping by using spatial facilitation. We tested the hypothesis that reflex pathways arising from two different cutaneous nerves converge on putative common INs in a spinal cord pathway for leg muscles during “reduced” locomotion. Furthermore, we hypothesized that the effect during combined stimulation of two nerves would be modulated according to the number of limbs engaged in arm and leg movement.

## Methods

### Subjects

Twelve subjects between the ages of 22 and 49 years participated with informed, written consent in a protocol approved by the Human Research Ethic Board at the University of Victoria and conforming to the Declaration of Helsinki (1964).

### General procedures

**Figure 1:**
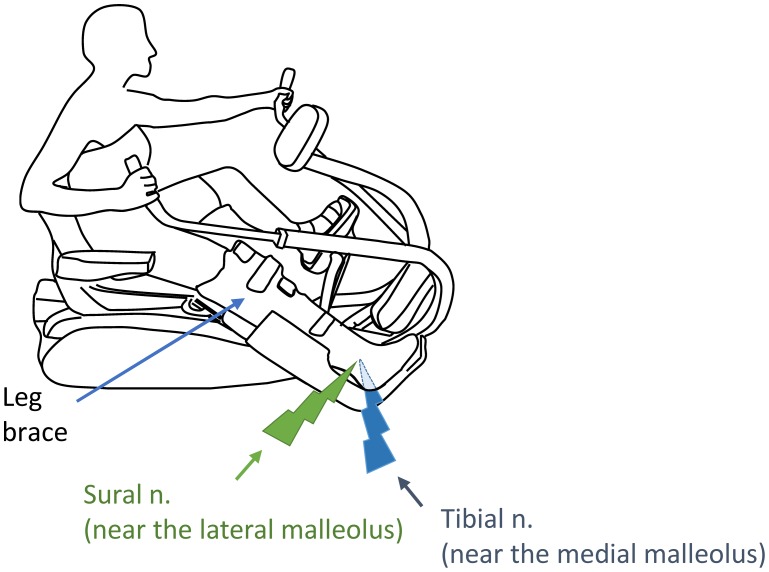
Experimental set-up for arm and leg remote rhythmic movement (ARM&LEG) on the recumbent stepping ergometer with assisted the arms. Nerve stimulation on the right leg was delivered at the start of the recovery phase of the left leg. Green lightning bolt: sural nerve stimulation (near the lateral malleus). Blue lightning bolt: distal tibial nerve stimulation (near the medial malleus).

**Figure 2:**
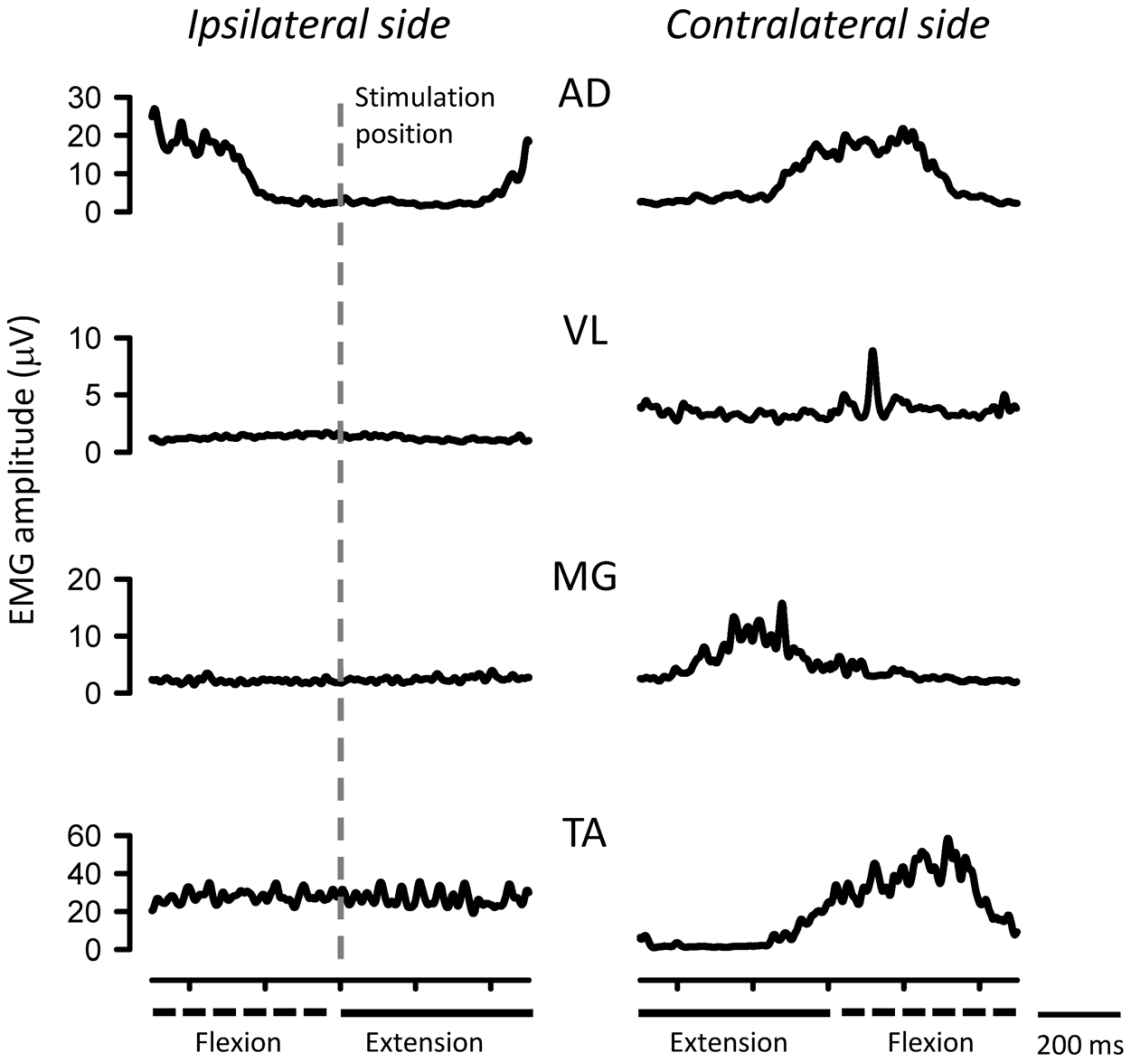
Typical recordings of EMG activities in AD, VL, MG and TA muscles during ARM&LEG movement for a single subject. Gray vertical line: stimulation position of ipsilataral side. Dashed lines: flexion phase of movement. Thick lines: extension phase of movement.

Participants used a recumbent arm and leg stepping ergometer (NuStep, TRS 4000, Ann Arbor, Michigan, USA) to perform the following rhythmic movement tasks: (1) bilateral arm and contralateral leg (ARM&LEG, Fig. 1); (2) bilateral arm (ARM); and, (3) contralateral leg (LEG). The right leg with nerve stimulation (ipsilateral or “test” leg) was fixed to a wooden platform to minimize any unwanted movement and a brace was worn to restrict right leg movement and fixed on the knee and ankle positions, respectively, at ∼160 and 90 degrees in all experiments (see Fig. 1). Movement frequency was maintained at ∼1 Hz during tasks [23]. Before experiments, participants practiced with auditory feedback of movement frequency using the metronome. While keeping the pace of these rhythmic movements with the feedback, subjects were asked to maintain a consistent isometric contraction (contraction level: ∼10% of EMG maximal value) of the ipsilateral TA muscle.

### Nerve stimulation

Cutaneous reflexes in TA were evoked with trains (5 pulses×1.0 ms at 300 Hz) delivered by an isolated constant current stimulation (Grass S88 stimulator with SIU and CCU1 units AstroMed-Grass Inc., Longueuil, Quebec, Canada). Stimulation was applied to the distal tibial (TIB; near the medial malleous), Sural (SUR; near the lateral malleous) and combined simultaneous stimulation of both nerves (TIB&SUR) (see Fig. 1). Stimulation was set as a multiple of the threshold at which a clear radiating paresthesia (radiating threshold, RT) into the innervation area of the nerve was reported.

### Stimulation procedures

Initially, subjects performed the ARM&LEG task in 3 different randomly ordered nerve stimulation conditions: 1) TIB, 2) SUR and 3) TIB&SUR. Stimuli were applied at near the start of the recovery phase of the contralateral leg by using the elbow or knee goniometer (Biometrics Ltd. Qwenfellinfach, UK) as a trigger signal (Fig. 1). After the ARM&LEG task, subjects were asked to perform ARM and LEG movement (each in separate trials) while combined stimulation was provided to the TIB&SUR nerves at the stimulation intensities used for each participant during ARM&LEG movement.

Stimulation intensities of SUR (∼1.4–2.2×RT) and TIB (∼0.8–2.2×RT) was adjusted to just below the threshold [just below 2 standard deviations (SDs) of the mean background TA EMG (BG EMG)] that produced an early latency (ELR latency: 45–80 ms after onset of stimulation) facilitatory reflex during the ARM&LEG task. These trains of stimuli were delivered every 2–4 step cycles for a total of 20 times in each trial and with a trial duration of ∼60 s. Furthermore, resting duration between trials was given at 3–5 min. To investigate convergence effects on ELR amplitude, simultaneous nerve stimulation (TIB&SUR) was applied using the identified subthreshold intensities.

### Electromyographic (EMG) recording

After abrasion and cleaning of the skin with alcohol, disposable surface EMG electrodes (Thought Technologies Ltd., Montreal, QC, Canada) were applied in a bipolar configuration with a 2 cm interelectrode distance over 8 muscles in the arms and legs. The muscles were anterior deltoid (AD), vastus lateralis (VL), medial gastrocnemius (MG) and tibialis anterior (TA). EMG signals were amplified (×5000) and bandpass filtered at 100–300 Hz (P511 Grass Instruments, Astro Med, Inc.).

### Data analysis

Data were sampled at 1000 Hz with a 12 bit A/D converter connected to a microcomputer running LabView Software (National Instruments, Austin, TX, USA). Evoked EMG in ipsilateral TA was analyzed for phasic reflex amplitudes and latencies. During offline digital processing using custom written MATLAB (Math Works Ink., Nantick MA) routines, the stimulus artifact was removed and the sweeps were then filtered with a dual pass Butterworth at 100 Hz. The peak response within the ELR window was determined and a 5 ms average centered around this peak was calculated. Reflex amplitudes were normalized as a percentage of the maximal EMG values while performing maximal voluntary contraction. These data from each participant were then averaged across all participants to obtain the group data.

### Statistics

To determine whether ELR amplitudes from TIB, SUR, simultaneous TIB&SUR stimulation and algebraic summation (TIB+SUR) were significantly modulated during ARM&LEG, one-way repeated measures analysis of variance (ANOVA) was performed. Also, comparison of reflex amplitudes and BG EMG activity following TIB&SUR stimulation across ARM&LEG, ARM, and LEG tasks were analyzed by using one-way ANOVA. Before calculating one-way ANOVA, Mauchly's sphericity test was performed as a test of the homoscedasticity of our samples [26].

If the results of ANOVA were statistically significant, multiple comparisons were performed with the Bonferroni post-hoc test [26].

All data are expressed as means and +/− SD. All repeated one-way ANOVA and post-hoc tests were performed using SPSS software Ver.11 (SPSS, Chicago, USA). Significant differences were recognized at the p<0.05 level in all cases.

## Results

### EMG patterns during ARM&LEG movement

Figure 2 shows typical recordings of EMG activities in muscles during ARM&LEG movement for a single subject. EMG activities of the AD, VL, MG, and TA on the contralateral side and ipsilateral AD were modulated during rhythmic movement. In contrast, EMG activity of the ipsilateral TA (“test” limb) remained constant. Activities of other ipsilateral leg muscles (VL and MG) were relatively inactive.

### Combined effect of simultaneous TIB&SUR nerve stimulation on ELR during ARM&LEG movement

**Figure 3:**
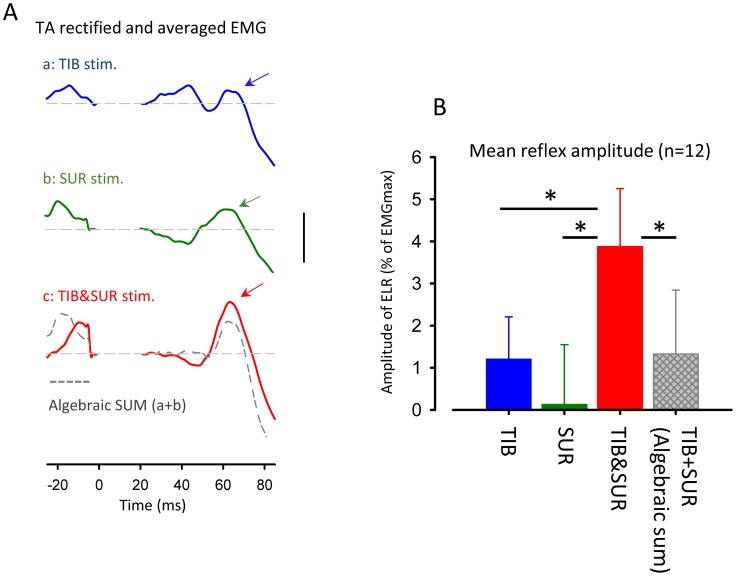
Convergence effect of simultaneous sural and tibial nerve stimulation on TA cutaneous reflex amplitude during ARM&LEG movement. (A) Full-wave rectified and averaged EMG in TA muscle following simultaneous combined stimulation of sural and tibial nerves (TIB&SUR, red trace), sural alone (SUR, green trace) and tibial alone (TIB, Blue trace) obtained from a single subject. Dashed gray trace: the mathematical summation of EMG traces for individual TIB and SUR nerves stimulation (Algebraic SUM). (B) Grand means (± SD) of the magnitudes of early-latency reflex responses (45–80 ms after stimulation onset) following simultaneous combined stimulation of sural and tibial nerves (Red bar), sural alone (Green bar) and tibial alone (Blue bar) obtained from 12 subjects. Hatched gray bar: mathematical summation of reflex amplitude for individual nerve stimulation (TIB+SUR). * *p*<0.001. Calibration bar = 10 µV.

Figure 3A illustrates typical recordings of cutaneous reflexes in TA muscle following TIB, SUR and simultaneous TIB&SUR stimulation while performing ARM&LEG in a single subject. ELR facilitation following simultaneous nerve stimulation was larger than that of SUR and TIB nerve stimulation alone. Interestingly, in this subject, ELR amplitude was enhanced during TIB&SUR compared with the mathematical summation of reflex responses for individual nerve stimulation (TIB+SUR; gray dashed trace).

Figure 3B shows mean amplitudes of ELR by stimulating TIB, SUR and TIB&SUR obtained from 12 subjects. Hatched gray bar indicates mathematical summation (TIB+SUR) of reflex amplitudes for individual nerves. Although the amplitudes of BG EMG activity in the TA were maintained relatively constant across stimulus conditions [TIB: 10.1±2.5%, SUR: 10.0±2.0% TIB&SUR: 10.5±2.6% of EMGmax (mean ± SD), one-way ANOVA: F(2,22) = 1.105 p>0.05], the mean amplitudes of TA reflexes were significantly enhanced in TIB&SUR (Bonferonni test; p<0.001). One-way ANOVA for comparing reflex amplitudes demonstrated a significant main effect [F(3,33) = 25.602 p<0.0001]. In the grouped data, the facilitation was significantly larger than expected based on mathematical summation of amplitudes for individual nerve stimulation (Bonferonni test; p<0.001). The non-linear summation of the inputs from TIB and SUR nerve stimulation were evident across all subjects (n = 12).

### Effect of the number of moving limbs on reflex amplitude

**Figure 4:**
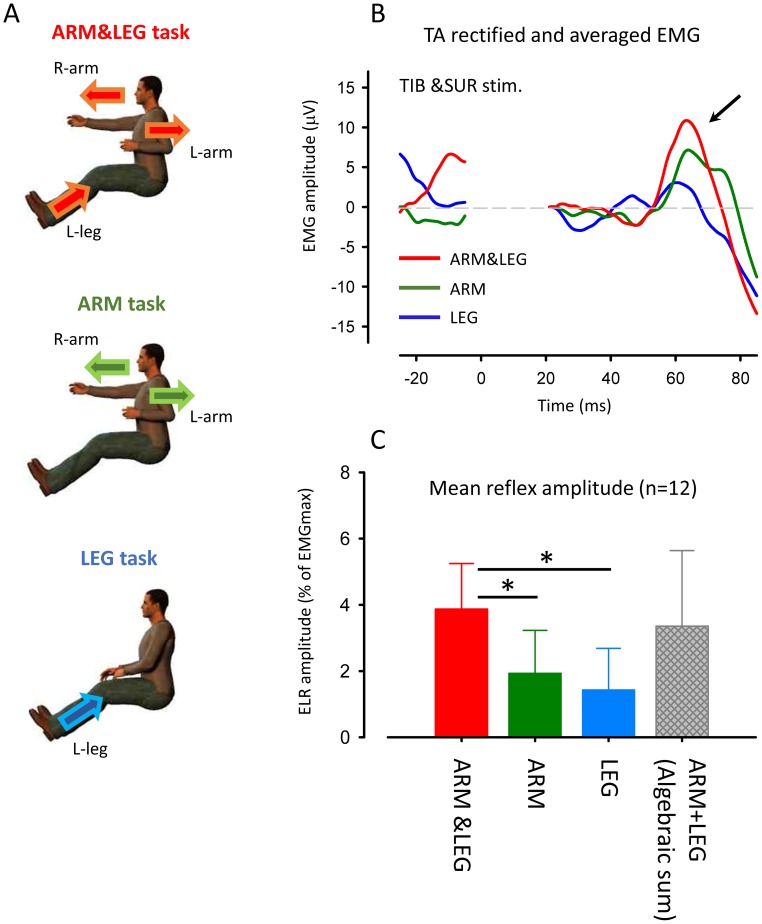
Effect of movement limbs on ELR following combined tibial and sural nerve stimulation (TIB&SUR). (A) Experimental tasks for remote rhythmic movements: arm and leg movement (upper panel, ARM&LEG), bilateral arm movement (middle panel, ARM) and contralateral leg movement (lower panel, LEG) (B) Full-wave rectified and averaged EMG in TA muscle following TIB&SUR stimulation during ARM&LEG (red trace), ARM (green trace) and LEG (blue trace) movement. (C) Grand means (± SD) of the magnitudes of early-latency reflex responses (45–80 ms after stimulation onset) following simultaneous combined stimulation of sural and tibial nerves during ARM&LEG (red bar), ARM (green bar) and LEG (blue bar) movement obtained from 12 subjects. Hatched gray bar: mathematical summation of reflex amplitude in individual tasks (ARM+LEG). * *p*<0.001.

To investigate whether the number of moving limbs modulates facilitation of ELR following simultaneous stimulation, data were compared across the ARM&LEG, ARM and LEG tasks (Fig. 4A).

Figure 4B shows typical recording of the TA cutaneous reflexes following TIB&SUR stimulation while performing ARM&LEG, ARM and LEG tasks for a single subject. These waveforms were obtained from the same subject whose data are presented in Fig. 3A.The facilitation of ELR seen during combined nerve stimulation was reduced while performing ARM and LEG compared to the ARM&LEG task (Fig. 4B).

Figure 4C illustrates mean ELR amplitudes by stimulating SUR, TIB and TIB&SUR obtained from 12 subjects. There was no significant differences in BG EMG activities in the TA muscle across ARM&LEG, ARM and LEG tasks [ARM&LEG: 10.5±2.6%, ARM: 10.1±2.7% LEG: 11.1±3.0% of EMGmax (mean ± SD), One-way ANOVA: F(2,22) = 2.785 p>0.05)]. One-way ANOVA for comparing reflex amplitudes demonstrated a significant main effect [F(3,33) = 18.757 p<0.0001]. Amplitudes of reflex responses, though, following SUR&TIB stimulation during ARM&LEG task were significantly greater than those during ARM and LEG movements (Bonfferoni test, p<0.001). The hatched bar represents the mathematical summation of reflex amplitudes during separate ARM and LEG movement tasks. Interestingly, there are no significant difference between reflexes of ARM&LEG and mathematical summation of ARM+LEG in the separate tasks (Bonfferoni test, p>0.05).

## Discussion

The present investigation demonstrated a non-linear facilitation of ELR amplitudes evoked by stimulating TIB and SUR nerves when activated simultaneously during rhythmic locomotor movement. During ARM&LEG movement, facilitation of ELR in TA following combined TIB&SUR stimulation was significantly larger than the amplitude anticipated by mathematical summation of amplitudes for individual nerve stimulation conditions (i.e. TIB+SUR). Interestingly, this extra facilitation seen during combined TIB&SUR was significantly reduced if subjects performed ARM or LEG tasks compared to the ARM&LEG task. An important advance of the present study was our ability to determine the incremental effect the number of rhythmically active limbs has on the excitability of common reflex pathways from sensory pathways innervating different foot regions.

### Non-linear facilitation of ELR during ARM&LEG movement

Stimulus intensities for individual nerve (TIB and SUR) were set to just below threshold for evoking significant ELR during ARM&LEG. Despite that, we found significant facilitation of ELR in TA following combined TIB&SUR stimulation. Moreover, this facilitation was larger than predicted by mathematical summation of responses following individual nerve stimulation. Thus, it is unlikely that our “extra facilitation” effect can be explained by the simple summation effects arising from excitability in “private reflex pathway” from inputs evoked by SUR and TIB stimulation [27]–[30]. In addition, there was no significant difference in BG EMG activities of the target TA muscle across nerve stimulation conditions. Thus, we suggest that background excitability of the motoneuronal pool remained relatively controlled across the three stimulation conditions.

The simplest interpretation of our results is that they are consistent with the concept of convergence onto shared interneurons within the polysynaptic reflex pathways arising from two different inputs described by Anders Lundberg and his co-workers [27], [28], [31]. In an elegant animal model, the convergence effect in cutaneous reflex pathways following simultaneous stimulation of two different nerves of the hindlimb was tested by Labella and McCrea (1990) using spatial facilitation in the anesthetized cat [15]. They observed that multiple cutaneous electrical stimulation of sural and femoral nerves elicited spatial facilitation of excitatory post synaptic potentials (EPSPs) in medial gastrocnemius motoneurons [15].

Although comparatively little is known about shared reflex pathways following low-threshold cutaneous nerves stimulation in humans, we found spatial facilitation in reflex pathways from cutaneous afferents innervating different foot regions during ARM&LEG movement [16]. These observations suggest the existence of shared reflex pathways to the human TA muscle while performing “reduced” locomotion. Thus, it is likely that reflex pathways from both nerves receive a similar facilitatory drive, for example from the flexor part of the locomotor pattern generator [2].

Interestingly, the range of ELR investigated in the present study is consistent with early latency or P1 components in the TA described by previous studies in the cat [32], [33]. Based on these latencies it is likely that spinal circuitry must be largely involved [*cf.* 32], [33].

Taken in sum, our findings suggest that locomotor commands and afferent feedback during ARM&LEG movement produce the neural substrate that facilitates transmission of ELR pathways during TIB&SUR nerve stimulation. We suggest that signals for rhythmic limb movement converge onto common reflex pathways coming from cutaneous afferents of different nerves innervating the foot.

### Activation of neural mechanisms in common reflex pathways during rhythmic locomotor movement depends on the number of moving limbs

We demonstrated that ELR amplitudes following TIB&SUR stimulation were strongly dependent on the task performed. That is, the amplitude of the ELR was significantly reduced when subjects performed ARM alone and LEG alone compared to ARM&LEG movement. This general feature of task dependency is in line with many previous reports obtained from individual nerve stimulation [2], [11], [12], [14], [20], [23], [25], [32], [34]–[37]. An extension to this literature found here is that there were no significant differences between reflexes during ARM&LEG movement and mathematical summation of both separate tasks (ARM alone or LEG alone).

**Figure 5:**
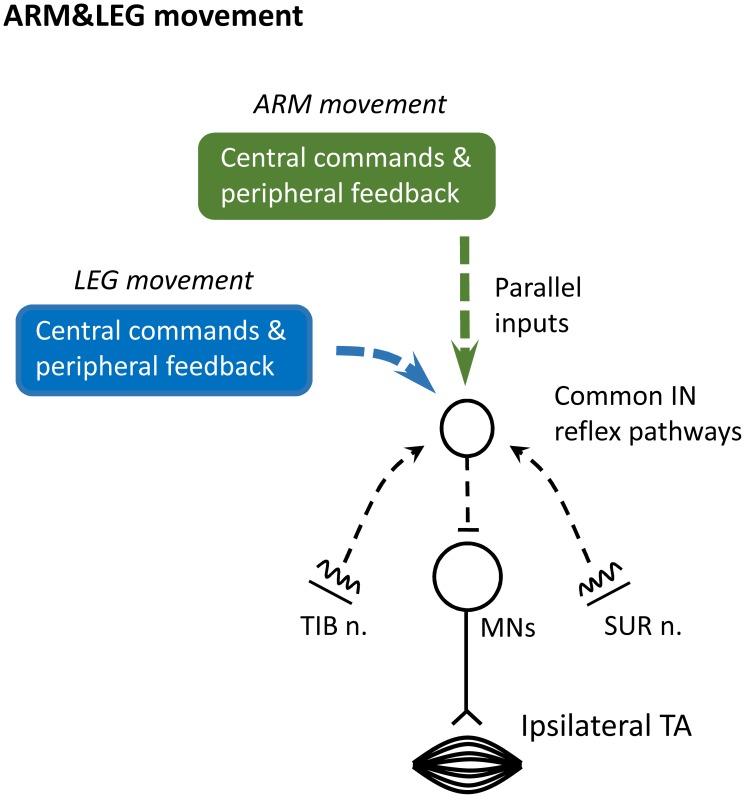
Schematic diagram outlining the possible neural pathways for integration of cutaneous reflex pathways inputs from different nerves of foot during arm and leg remote rhythmic movement (ARM&LEG). At the center is a simplified interneuronal (IN) reflex pathways in tibialis anterior muscles. Inputs from the sural and tibial nerves have connections onto shared INs (dashed black lines). Excitability of common INs and motoneurons (MNs) are regulated by central commands and peripheral feedback related to LEG (blue square) and ARM (green square) movement. It is possible that individual input arising from each LEG and ARM movement converge “in parallel” onto these putative reflex pathways (dashed lines of blue and green squares) during ARM&LEG movement. TIB n.: tibial nerve. SUR n.: sural nerve. MNs: motoneurons.

To provide a context for our findings, we propose a schematic representation of the possible neural mechanisms as shown in Figure 5. This figure is an admitted oversimplification of the likely set of connections in the human spinal cord but it can be a useful approximation for discussing our findings and for framing additional research questions.

Figure 5 shows reflex pathways with cutaneous inputs evoked by stimulation of TIB nerve and SUR nerve during ARM&LEG movement. These two inputs (dashed black arrows) are shown projecting to common IN reflex pathways sketched by the small open circle. In our study, observation of non-linear ELR facilitation following combined SUR and TIB nerves stimulation infers the existence of these putative converging pathways (*c.f.* Fig. 3A and B) [*cf.* 28]. Thus, the simplest explanation is that individual inputs arising from activity during LEG and ARM movement converged onto “parallel” convergent pathways during ARM&LEG movement (see squares of blue and green). Although other contribution cannot be denied, we believe that this explanation is the simplest and most reasonable interpretation based on our observations.

### Translational applications for rehabilitation

We found that rhythmic locomotor movement activates presumed common reflex pathways from sensory inputs innervating different foot regions. In addition, our results suggest that transmission in these reflex pathways is weighted according to the number of limbs directly engaged in human locomotor activity. Sensory information modulates motor output in locomotor generator systems in the spinal cord [38]–[43]. After stroke and spinal cord injury (SCI), it has been suggested that these circuits need to be strongly activated for rehabilitation of walking abilities [18],[44]–[48]. More recently, Hubli & Dietz [48] reported that there was a correlation between walking ability and short latency reflex responses behavior in chronic SCI. As a translational implication for rehabilitation we suggest that stimulation of multiple nerves during arm and leg movement may be beneficial to improve access to IN circuits that interconnect locomotor regions in the human spinal cord. This observation could be used to enhance development of rehabilitative interventions as new strategies for recovery of walking abilities using arm and leg movement and sensory modulation from the hands and feet.
